# PrimaDORAC: An improved Web Interface for Rapid GAFF2 Parameter Assignment with ABCG2 Charge Models for Drug Design Applications

**DOI:** 10.1002/jcc.70444

**Published:** 2026-06-18

**Authors:** Piero Procacci

**Affiliations:** ^1^ Department of Chemistry University of Florence Florence Italy

## Abstract

The accurate parameterization of drug‐like molecules is a fundamental prerequisite for molecular dynamics simulations in structure‐based drug design. While the AM1‐BCC charge model has served as the *de facto* “standard” for GAFF2 parameterization for two decades, the recently introduced ABCG2 model offers substantially improved accuracy in predicting hydration free energies and other key physicochemical properties. However, accessing ABCG2 parameters traditionally requires downloading and installing the full AmberTools suite—a multi‐gigabyte software package with complex dependencies—presenting a significant barrier for many practitioners, particularly experimental collaborators and researchers new to the field. Here we present a major upgrade to the PrimaDORAC web interface that makes ABCG2 parameterization readily accessible to the entire drug design community. Through a minimalistic integration of essential AmberTools components directly into the web application framework, users can now obtain GAFF2 parameters with ABCG2 charges for any drug‐like molecule in seconds, without any software installation. The interface accepts a single SMILES string or structure file and returns a complete archive containing GROMACS‐compatible topology files and a ready‐to‐use PDB structure. By removing all technical barriers to accessing state‐of‐the‐art ABCG2 parameters, the upgraded PrimaDORAC interface is aimed at empowering medicinal chemists, pharmacologists, and computational researchers alike to conduct more accurate MD simulations with minimal effort. The service is freely available at www1.chim.unifi.it/orac.

## Graphical abstract

A web‐enabled implementation of the ABCG2 charge model within the PrimaDORAC platform provides frictionless GAFF2 ligand parameterization. Systematic chemical‐space analysis reveals class‐dependent correlations and structured deviations relative to AM1‐BCC, facilitating informed adoption of next‐generation electrostatic models in computational drug design.

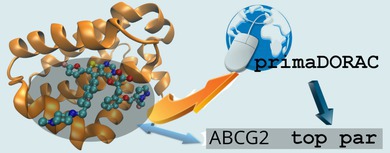

## 1 Introduction

The accurate parameterization of small organic molecules and drug candidates represents a critical prerequisite for molecular dynamics (MD) simulations of ligand‐protein systems. Among the various force fields available for such simulations, the Generalized Amber Force Field (GAFF) and its 2nd generation derivative GAFF2 have emerged as one of the most widely adopted choices due to their broad coverage of organic chemistry space and their demonstrated reliability in both hydration free energy calculations and protein‐ligand binding studies [1, 2]. A key component of GAFF/GAFF2‐based parameterization is the assignment of partial atomic charges, with the RESP (Restrained Electrostatic Potential) model as the default recommended method (quantum mechanics (QM) calculations are needed), and the AM1‐BCC (Austin Model 1‐Bond Charge Corrections) method representing the popular approach by users in practice for nearly two decades [3, 4].

The AM1‐BCC methodology, originally developed by Jakalian and coworkers, combines semi‐empirical AM1 calculations with empirical bond charge corrections to reproduce high‐quality restrained electrostatic potential (RESP) charges at a fraction of the computational cost [3]. This approach has been extensively validated and has become the default charge model for GAFF2 parameterization in numerous software packages and workflows [5, 6, 7, 8]. However, despite its widespread adoption, the AM1‐BCC model exhibits limitations in predicting certain molecular properties, particularly hydration free energies, where root‐mean‐square errors (RMSE) of approximately 1.7 kcal/mol have been reported on benchmark datasets such as FreeSolv [9, 10].

Recently, Junmei Wang and coworkers [11] introduced a significant advancement in charge parameterization with the development of the ABCG2 (AM1‐BCC‐GAFF2) charge model. By re‐optimizing the bond charge correction terms for specific functional groups against high‐quality experimental hydration free energy data, the ABCG2 model achieves substantially improved accuracy, lowering the RMSE to approximately 1.0 kcal/mol on the same FreeSolv benchmark. This represents a nearly 40% reduction in prediction error, demonstrating the potential of property‐specific force field optimization. Subsequent studies have confirmed the superior performance of ABCG2 not only for hydration free energies but also for transfer free energies of solutes from water to organic solvents [12].

Despite the clear advantages of the ABCG2 model, its adoption by the computational chemistry community faces a significant practical barrier. The parameterization tools required to generate ABCG2 charges—specifically the “antechamber”, “atomtype”, “bondtype”, “am1bcc”, and “parmchk2” programs—are distributed as part of the AmberTools suite, a comprehensive software package exceeding several gigabytes in size [13]. While AmberTools is freely available, its installation typically requires users to navigate complex dependencies, often via Miniconda or similar package management systems, and involves downloading the entire suite even when only the charge assignment functionality is needed.

Several web‐based solutions have been developed to address the accessibility challenge in molecular parameterization. Tools such as LigParGen [14] and Acpype [15] provide OPLS‐AA and AM1‐BCC parameter assignment, respectively, while more recent platforms like OpenFE offer comprehensive free energy perturbation setup services [16], including force field parameteriztion of ligands. However, these solutions often target specific force fields or simulation workflows and may not provide direct access to the latest charge models within the GAFF2 framework. The original PrimaDORAC web interface, introduced in 2017, addressed this gap by providing a simple, freely accessible platform for GAFF2 parameter assignment using AM1‐BCC charges [17]. PrimaDORAC demonstrated remarkable reliability, successfully parameterizing over 52,000 compounds from the National Cancer Institute Open Database and producing minimized structures with mean root square displacements of only 0.01–0.02 nm relative to reference geometries [18].

In this work, we present a significant upgrade to the PrimaDORAC web interface that extends its functionality to include the novel ABCG2 charge model alongside the traditional AM1‐BCC method and the original PrimaDORAC assignment protocol. This upgrade has been achieved through a minimalistic porting and integration of the essential AmberTools components directly into the PrimaDORAC web application framework. By extracting only the code modules required for charge assignment and bonded parameter generation, we have created a lightweight implementation that preserves all functionality while eliminating the need for users to download and install the full multi‐gigabyte AmberTools distribution.

The upgraded PrimaDORAC interface offers three distinct charge parameterization pathways, namely the ABCG2 newly implemented method, the still widely used AM1‐BCC model, and the legacy parameterization protocol from the initial release [17]. Users simply provide a SMILES string or upload a molecular structure file, select their preferred charge model and optimization options, and within seconds receive a downloadable archive containing GROMACS‐compatible “.itp” topology files and a corresponding “.pdb” coordinate file, ready for immediate use in MD simulations. This streamlined workflow eliminates all software installation requirements, making state‐of‐the‐art GAFF2 parameterization with the latest ABCG2 charges accessible to anyone with an internet connection.

By incorporating the ABCG2 model as the default choice, our upgraded interface now provides access to a charge parameterization that has been demonstrated to outperform AM1‐BCC in multiple property predictions while maintaining comparable accuracy in protein‐ligand binding free energy calculations [19].

In the remainder of this paper, we briefly describe the technical implementation of the upgraded interface, present a comprehensive comparative analysis of AM1‐BCC and ABCG2 charges across diverse chemical classes using a systematically curated SMILES test set that spans multiple functional group families, and discuss the implications for drug design applications. Our results demonstrate that while both charge models yield broadly similar charge distributions, significant differences emerge for heteroatoms (nitrogen, oxygen, sulfur, phosphorus) and the hydrogen and carbon atoms directly bonded to them, providing valuable insights for practitioners selecting charge models for specific chemical classes.

## 2 Methods

**FIGURE 1:**
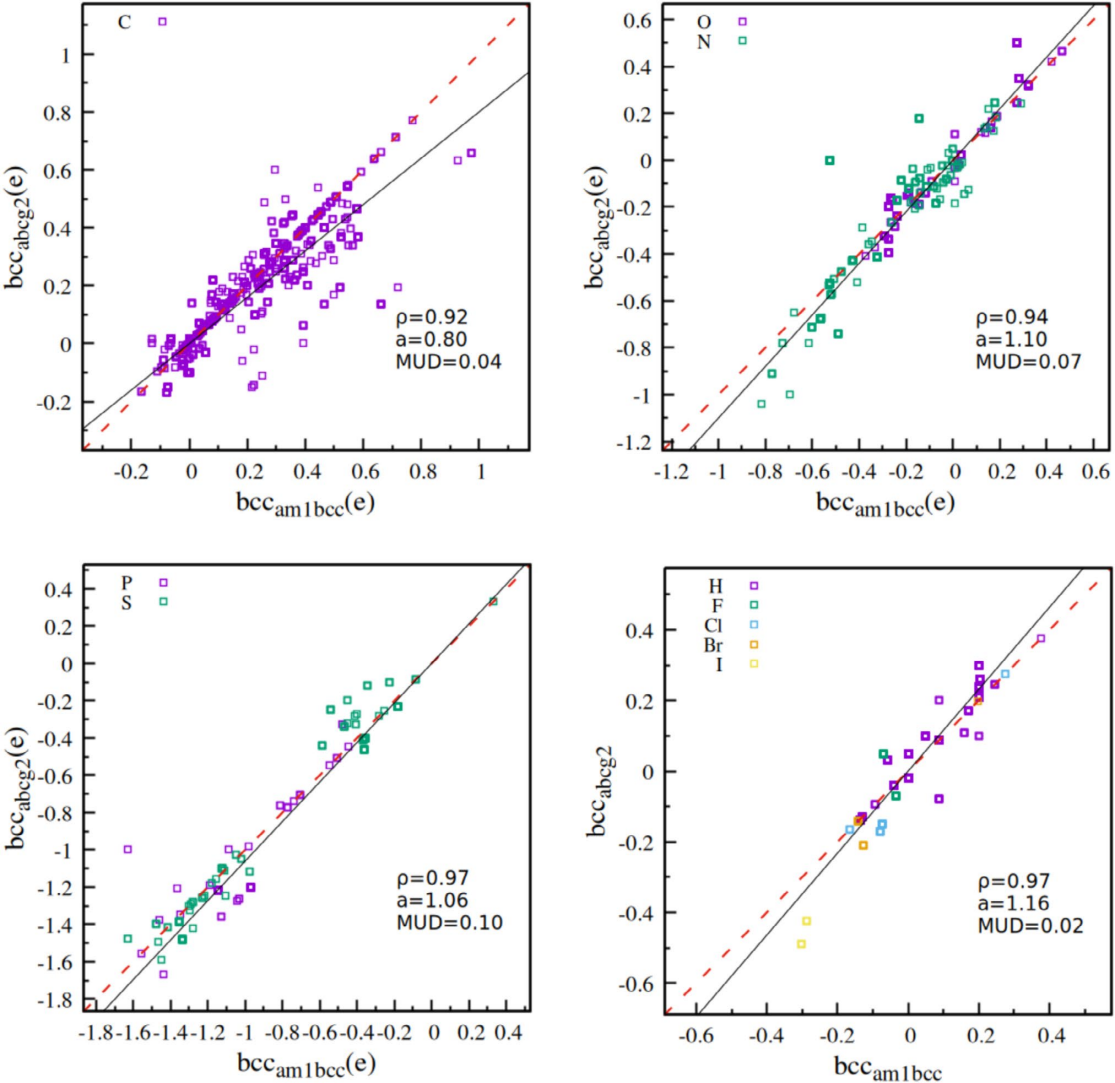
Scatter plots comparing AM1‐BCC and ABCG2 bond charge corrections for different atom types. Top left: carbon atoms; top right: nitrogen and oxygen atoms; bottom left: phosphorus and sulfur atoms; bottom right: hydrogen and halogen atoms. Data are extracted from the compound set described in the text (SMILES codes available in the Zenodo repository, https://zenodo.org/records/18837123). The red dotted line indicates perfect correlation (y=x), while the black line represents the best linear fit to the data.

The upgrade to include the ABCG2 parameterization in PrimaDORAC was implemented by integrating essential AmberTools components into the existing Fortran 90 main parser of the interface (see the flow chart in Figure 1 of reference [17]). In this revised parser, atom type assignment and bond charge corrections (BCC) are now managed through calls to the antechamber, am1bcc, and parmchk2 programs. The BCC for each atom is derived by subtracting the Gasteiger charges, computed by antechamber using the “‐c gas” option, from the post‐BCC charges provided by the am1bcc program. Consistent with the original PrimaDORAC release, the full AM1‐BCC or ABCG2 charges are obtained by summing the AM1 Mulliken charges and the BCC. Upon successful completion, the interface delivers the ligand parameterization in prm/tpg format for the ORAC program [20] and as itp/top files for the GROMACS code [5]. Additionally, gas‐phase IR intensities, as predicted by the chosen force field (ABCG2, AM1‐BCC, or the legacy PrimaDORAC method), are included in the output archive for the ligand.

Similar to other web‐based parameterization tools [14, 15], PrimaDORAC processes a single input SMILES string or uploaded file per session. For batch processing of SMILES lists or PDB/MOL2/SDF files, users can employ the primadorac.bash script, which is distributed with the ORAC 6.3 source code under the GNU General Public License v3. As an illustration, the following bash script generates AM1‐BCC and ABCG2 tpg/prm files for all SMILES codes listed in a file named list.smi:#!/bin/bash j=0 for i in ‘cat list.smi‘; do.  ((j++)).  echo $i | awk’{print $1}’ > tmp.smi.  obabel -ismi tmp.smi -opdb -Otmp.pdb --gen3D >& out.   cp tmp.pdb ligA$j.pdb.  cp tmp.pdb ligJ$j.pdb.  primadorac.bash -pA ligA$j.pdb.  primadorac.bash -pJ ligJ$j.pdb.done


The ‐pA and ‐pJ options direct the primadorac.bash script to generate parameters and atom types for pH 7 using the ABCG2 and AM1‐BCC models, respectively. This process relies on AM1‐optimized geometries and charges computed with MOPAC. The complete ORAC distribution is provided as a lightweight 50 MB tar.gz archive. ORAC can be installed on any Unix‐like system by following the building instructions on the ORAC website, hosted at the University of Florence (www1.chim.unifi.it/orac). In addition to the ORAC executable, the primadorac.bash script requires widely available standard tools, including gfortran, OpenBabel [21], and MOPAC [22, 23].

## 3 BCC Comparison for the AM1‐BCC and ABCG2 Models

In a recent benchmark study [11], Junmei Wang and coworkers systematically compared the AM1‐BCC and ABCG2 models by evaluating hydration free energies and octanol–water transfer free energies across extensive datasets of small organic molecules, including the FreeSolv and Minnesota solvation databases [9, 24]. Their results demonstrated a decisive improvement in accuracy when employing the ABCG2 model. Here, we seek to rationalize the molecular origins of these macroscopic differences by analyzing the bond charge corrections (BCCs) themselves—the sole source of discrepancy between the two charge models—across a diverse and chemically curated set of compounds.

Our analysis encompasses approximately 400 molecules, spanning nine chemically homogeneous categories that collectively represent the structural and functional diversity of modern drug‐like compounds. The full list of SMILES codes, along with the corresponding BCC values computed with both AM1‐BCC and ABCG2, is available in the Zenodo repository (https://zenodo.org/records/18837123).

It is important to note that AM1‐BCC and ABCG2 share identical underlying foundations: both models rely on the same GAFF2 parameter set (gaff2.dat, version 2.2.20, March 2021, distributed with AmberTools) and both employ the same core Mulliken charges obtained from semiempirical AM1 calculations on SQM‐ or MOPAC‐optimized geometries. Consequently, for any given molecule, the atom types and the core Mulliken charges are identical under both parameterizations. Differences in the final atomic charges arise exclusively from the distinct BCCs assigned by each model. This equivalence isolates the BCCs as the unique source of variation, making them the ideal focus for a comparative analysis.

In Table 1, we quantify the differences between the AM1‐BCC and ABCG2 BCC across the nine compound classes. For each class, we report the Pearson correlation coefficient (ρ), the slope (a) and intercept (b) of the best‐fit linear regression, the mean unsigned deviation (MUD), the Kendall rank coefficient (τ), and the total number of atoms (n) included in the correlation.

**TABLE 1:** Correlation metrics between the total atomic bond charge obtained with the AM1‐BCC and ABCG2 models.

Type	ρ	a	b	MUD (e)	τ	n
CARB	0.85	0.91	0.00	0.01	0.56	113
OXY	0.96	0.92	0.00	0.02	0.78	198
NITR	0.98	1.08	0.00	0.03	0.77	377
HALO	0.89	0.94	0.00	0.04	0.62	616
PHOS	0.96	1.02	0.00	0.04	0.83	1279
SULF	0.99	1.00	0.00	0.03	0.79	1542
HETE	0.92	0.79	0.00	0.04	0.61	543
WARH	0.95	1.04	0.00	0.04	0.54	379
BIOL	0.96	0.99	0.00	0.03	0.80	894

Abbreviations: a, b: slope and intercept of the best‐fit line; τ: Kendall rank coefficient; ρ: Pearson correlation coefficient; n: total number of atoms included in the correlation; BIOL: biologically relevant molecules and drug fragments; CARB: fundamental hydrocarbons; HALO: halogenated compounds; HETE: heterocyclic compounds; MUD: mean unsigned BCC deviation (in units of electrons); NITR: nitrogen‐containing functional groups; OXY: oxygen‐containing functional groups; PHOS: phosphorus‐containing compounds; SULF: sulfur‐containing compounds; WARH: compounds with covalent warheads (nitriles and alkynes).

For all compound classes, the intercept b is identically zero, and the mean signed deviation (MSD, not shown) also vanishes. This is an important internal consistency check for the PrimaDORAC implementation: because both models originate from identical Mulliken core charges and differ only in their BCC contributions, conservation of the total molecular charge demands that switching from AM1‐BCC to ABCG2 introduces no systematic global offset. The vanishing intercept and MSD therefore confirm that total charge is rigorously preserved upon migrating to the ABCG2 parameterization.

The slope a provides direct information on systematic magnitude scaling between the two BCC sets. A slope deviating from unity indicates a multiplicative bias in the ABCG2 corrections relative to AM1‐BCC. For example, a=1.08 for nitrogen‐containing compounds (NITR) implies that ABCG2 BCC magnitudes are, on average, 8% larger than their AM1‐BCC counterparts. Conversely, a=0.91 for hydrocarbons (CARB) indicates that ABCG2 corrections are approximately 9% smaller. Notably, for saturated hydrocarbons, the BCCs are identical between the two models; the observed differences in the CARB class arise exclusively from hydrogen atoms bonded to aromatic carbons (ha atom type), which receive non‐zero BCCs only in the ABCG2 model.

The most pronounced scaling deviation is observed for heterocycles (HETE), with a=0.79, indicating a substantial rescaling. ABCG2 BCC magnitudes are, on average, 21% smaller than those of AM1‐BCC. This finding is particularly significant given the prevalence of heteroaromatic rings in drug‐like molecules and their critical role in establishing electrostatic interactions with protein targets. In contrast, sulfur‐ and phosphorus‐containing compounds (SULF, PHOS) exhibit slopes very close to unity, suggesting near‐equivalence in the overall magnitude of the BCC corrections for these elements.

The Pearson coefficient ρ measures the strength of linear correlation and reflects the overall agreement in variance between the two BCC sets. Values approaching unity (e.g., SULF: 0.99, NITR: 0.98, PHOS: 0.96) indicate that the two BCC sets are nearly perfectly linearly related, differing primarily by small scaling factors. Lower values (e.g., CARB: 0.85, HALO: 0.89) suggest increased scatter and a less uniform mapping between the two parameterizations, implying that for these classes the relationship between AM1‐BCC and ABCG2 corrections is more complex than a simple scaling transformation.

The mean unsigned deviation (MUD) quantifies the average absolute difference between the BCC values. Across all categories, MUD remains modest (0.01–0.04 e), confirming that the two charge models remain quantitatively close at the atomic level. However, even these seemingly small differences can have non‐negligible consequences for electrostatic potentials, particularly in regions of tight steric confinement, such as protein binding pockets. Notably, halogenated compounds (HALO) and heterocycles (HETE) exhibit the largest MUD values (0.04 e), consistent with the pronounced scaling deviations noted above.

The Kendall rank coefficient τ assesses whether the ordering of BCC magnitudes is preserved between the two models. While Pearson ρ captures linear variance agreement, τ probes rank consistency—an important metric when the absolute values of BCCs are less critical than their relative ordering, as in comparative binding studies. High τ values (e.g., PHOS: 0.83, BIOL: 0.80, SULF: 0.79) indicate that the two models not only scale similarly but also largely preserve the relative ranking of individual bond corrections. Lower values (e.g., WARH: 0.54, CARB: 0.56, HALO: 0.62) suggest local reordering effects, which may be chemically meaningful in reactive environments (covalent warheads) or in electronically heterogeneous regions where subtle differences in charge distribution can influence reactivity and binding.

In summary, the metrics presented in Table 1 paint a coherent picture: ABCG2 can be viewed, for most chemical classes, as a refined and selectively rescaled version of AM1‐BCC, incorporating class‐dependent magnitude adjustments and modest but non‐negligible rank reshuffling. The largest systematic differences emerge in heterocycles and nitrogen‐rich environments—precisely the chemical domains where electrostatic fidelity is most critical for accurate prediction of solvation thermodynamics and protein‐ligand interactions. These observations align with and rationalize the superior performance of ABCG2 reported in recent benchmarks, and underscore the value of making this advanced charge model readily accessible through the upgraded PrimaDORAC web interface.

Figure 1 presents scatter plots comparing the total atomic BCCs for carbon, nitrogen, oxygen, phosphorus, sulfur, hydrogen, and halogen atoms, computed using the AM1‐BCC and ABCG2 models on the same compound set analyzed in Table 1. The number of atoms of each type and their average BCC values are reported in Table 2.

**TABLE 2:** Atom counts per type and average atomic BCCs (in units of electrons) for the 388 compounds analyzed in this study.

	H	C	N	O	P	S	Halogens
n	2673	2088	322	595	68	122	70
$\left\langle \Delta q_{\mathrm{AM}1\mathrm{BCC}}\right\rangle$	0.0096	0.1299	−0.3410	−0.0354	−1.0207	−0.7639	−0.0846
$\left\langle \Delta q_{\text{ABCG}2}\right\rangle$	0.0119	0.1208	−0.3381	−0.00544	−1.1069	−0.7632	−0.1050

*Note:* The full dataset, including SMILES codes and computed BCC values, is available in the Zenodo repository (https://zenodo.org/records/18837123).

### 3.1 Carbon Atoms

Carbon atoms, which can receive positive or negative BCC contributions from up to four bonded neighbors, exhibit the smallest slope among the four panels (a=0.80). This indicates that, on average, BCC charges on carbon are scaled down by approximately 20% when switching from AM1‐BCC to ABCG2. The scatter around the best‐fit line is moderate (ρ=0.92), reflecting some variability in how the two models distribute BCCs among chemically distinct carbon environments. The relatively low mean unsigned deviation (MUD = 0.04 e) arises because the overall range of BCC variation for carbon atoms is narrower than that observed for more electronegative elements such as nitrogen and oxygen.

### 3.2 Nitrogen and Oxygen Atoms

For nitrogen and oxygen atoms, the data points are tightly clustered around the best‐fit line (ρ=0.94, a=1.10), consistent with the strong correlations observed for nitrogen‐ and oxygen‐containing compound classes in Table 1. The slope of 1.10 indicates an average 10% upscaling of BCC magnitudes for these electronegative atoms when using ABCG2. The MUD (0.07 e) is notably higher than for carbon, reflecting the wider intrinsic range of BCC values on highly polar N and O atoms. An interesting feature of the scatter plot is the apparent lower density of points for oxygen compared to nitrogen, despite oxygen atoms being more abundant in the dataset (595 O atoms vs. 322 N atoms, Table 2). This apparent discrepancy arises because many oxygen atoms are *terminal* (e.g., in carbonyl or hydroxyl groups) and receive BCC contributions from only one bond. Consequently, numerous oxygen atoms share identical or nearly identical BCC values, leading to degenerate points in the scatter plot. Nitrogen atoms, by contrast, are more frequently embedded in ring systems or involved in multiple bonds, yielding a wider distribution of BCC values and thus more distinct points.

### 3.3 Phosphorus and Sulfur Atoms

Consistent with the results in Table 1, phosphorus and sulfur atoms display the highest Pearson correlation coefficient (ρ=0.97) and a near‐unity slope (a=1.06), indicating only a modest 6% average upscaling from AM1‐BCC to ABCG2. The MUD (0.10 e) is the largest among all atom types, reflecting the exceptionally wide range of BCC variation for these elements, spanning more than 2 e from strongly negative to slightly positive values. Notably, the data points for phosphorus and sulfur cluster into two distinct groups. Strongly negative BCCs (below −1.0 e) correspond to atoms in high oxidation states (e.g., Amber atom types p4, p5 for phosphorus and s4, s6 for sulfur) (these BCCs are aimed at taming the high Mulliken positive charge from AM1 calculations), while BCCs in the range $\left[-0.5,0.2\right]$ e are characteristic of lower oxidation states where the atom receives BCC contributions from fewer than three bonds. This bimodal distribution reflects the diverse chemical roles of phosphorus and sulfur in drug‐like molecules, from highly polar phosphonates and sulfonates to more neutral thioethers and phosphines.

### 3.4 Hydrogen and Halogen Atoms

The bottom right panel of Figure 1 shows the correlation for terminal atoms—hydrogen and halogens (F, Cl, Br, I). Despite these atoms constituting more than 50% of the total atoms in the dataset (2673 H atoms plus 70 halogens, Table 2), the scatter plot reveals only approximately 20 distinct (degenerate) points. This degeneracy arises because hydrogen and halogen atoms are invariably terminal, receiving BCC contributions from a single‐bonded neighbor; consequently, their BCC values are determined almost exclusively by the atom type of the bonded heavy atom and are highly reproducible across chemically similar environments. The slope of 1.16 indicates an average 16% upscaling of BCC magnitudes for these terminal atoms when using ABCG2, while the near‐perfect correlation (ρ=0.97) and minimal scatter confirm the highly systematic nature of this scaling.

## 4 Conclusions

In this work, we have transformed the PrimaDORAC web interface into an effortlessly accessible platform for GAFF2 parameterization with the latest ABCG2 charge model. The ABCG2 model, as demonstrated by Junmei Wang and coworkers, reduces errors in hydration free energy predictions by nearly 40% compared to the long‐standing AM1‐BCC standard. For practitioners engaged in lead optimization, fragment‐based drug design, or binding affinity predictions, this improvement translates directly into more reliable simulations and better‐informed decisions. Yet, until now, accessing these improved parameters meant navigating the installation of AmberTools—a non‐trivial undertaking that has undoubtedly discouraged many potential users. Our minimalistic integration of the essential AmberTools components removes this barrier entirely, requiring nothing more than a web browser and an internet connection.

Our systematic comparison of AM1‐BCC and ABCG2 bond charge corrections across ≃ 400 chemically diverse compounds provides practical guidance for the community. The strong overall correlation between the two models (Pearson R>0.95) reassures practitioners that legacy AM1‐BCC parameters remain reasonable for many applications. However, the significant differences observed for heteroatoms and their directly bonded neighbors—precisely the atoms that dominate ligand recognition and solvation—highlight why ABCG2 achieves superior performance in thermodynamic property predictions. For medicinal chemists optimizing a series of compounds, these differences at key functional groups can profoundly influence calculated solvation energies and, by extension, predicted binding affinities.

Perhaps most importantly, this upgrade embodies a philosophy that advanced computational tools such as the ABCG2 model *should be accessible to all researchers, regardless of their technical expertise*. Similarly to the LibParGen and Acpype interfaces, PrimaDORAC lowers the barrier to rigorous computational work across the entire drug design community by eliminating the “installation tax” that has historically limited access to such tools.

## Conflicts of Interest

The author declares no conflicts of interest.

## Data Availability

For readers wishing to perform further analyses or reproduce the results presented here, the complete dataset—including the full list of SMILES codes, computed BCC values for both charge models, and the software tools used for analysis—is available in the Zenodo repository (https://zenodo.org/records/18837123). This resource also includes the scripts required to regenerate all figures and tables presented in this work, facilitating transparent and reproducible research.
